# Integrated Metabolomics and Transcriptome Analysis of Flavonoid Biosynthesis in Safflower (*Carthamus tinctorius* L.) With Different Colors

**DOI:** 10.3389/fpls.2021.712038

**Published:** 2021-07-26

**Authors:** Rui Wang, Chaoxiang Ren, Shuai Dong, Chao Chen, Bin Xian, Qinghua Wu, Jie Wang, Jin Pei, Jiang Chen

**Affiliations:** ^1^State Key Laboratory of Southwestern Chinese Medicine Resources, Chengdu University of Traditional Chinese Medicine, Chengdu, China; ^2^College of Pharmacy, Chengdu University of Traditional Chinese Medicine, Chengdu, China; ^3^The State Bank of Chinese Drug Germplam Resources, Chengdu University of Traditional Chinese Medicine, Chengdu, China

**Keywords:** safflower, color, metabolomics, transcriptome, C-glucosylquinochalcones, UGTs, P450

## Abstract

Safflower is widely used in dying and in traditional medicine, and C-glucosylquinochalcones are the main metabolic species in the red color of safflower. Various safflower cultivars have flowers with different colors. However, the metabolic and transcriptional differences among safflower cultivars with different-colored flowers and the genes participating in C-glucosylquinochalcone biosynthesis are largely unknown. To provide insights on this issue, we performed integrated metabolomics and transcriptome analyses on the flavonoid biosynthesis of flowers of different colors in safflower (white-W, yellow-Y, light red-LR, and deep red-DR). The metabolic analysis showed that flavonoid metabolites showed great differences among the different colors of safflower. More flavonoid metabolic species were detected in Y and W, while C-glucosylquinochalcones were not detected in W. The content of C-glucosylquinochalcones increased with increasing color. Transcriptional analysis showed that most of the annotated flavonoid biosynthesis genes were significantly increased in W. The expression of genes related to flavonoid biosynthesis decreased with increasing color. We analyzed the candidate genes associated with C-glucosylquinochalcones, and an integration of the metabolic and transcriptional analyses indicated that the differential expression of the chalcone synthase (CHS) gene is one of the main reasons for the difference in flavonoid species and content among the different colors of safflower. Combined with the expression pattern analysis, these results indicated that HH_035319, HH_032689, and HH_018025 are likely involved in C-glucosylquinochalcones biosynthesis. In addition, we found that their expression showed greatly increased after the methyl jasmonate (MeJA) treatment. Therefore, HH_035319, HH_032689, and HH_018025 might participate in C-glucosylquinochalcone biosynthesis, which ultimately leads to the red color in safflower.

## Introduction

Safflower (*Carthamus tinctorius*) is a member of the Asteraceae or Compositae family and represents an important commercial crop cultivated as an oilseed livestock feed or a dye source or for medicinal purpose (Ekin, 2005). This plant was likely domesticated in the fertile Mediterranean coastal zone over 4,000 years ago (Chapman and Burke, 2007). In the Compendium of Materia Medica, Zhang Qian introduced safflower to China during the Han Dynasty on a diplomatic mission to the Western Regions (*via* the Silk Road). Thus, safflower has been cultivated and used in China for more than 2,000 years. Currently, safflower has been widely cultivated in Asia, Europe, Australia, and the Americas. According to the United Nations Food and Agriculture Organization (FAO)^1^, 717,900 hectares of safflower were sown globally, which produced 666,600 tons of safflower in 2018. The flowers of safflower are mainly used as dyes and cosmetics worldwide (Azami et al., 2019), while they are used in traditional Chinese medicine as a medicine to improve cerebral blood flow and to treat various ailments, such as gynecological, cerebrovascular, and cardiovascular diseases, hypertension, and coronary heart disease (Lou and Liu, 1956; China T.S.P.Co, 2015).

In most plants, the colors of the flowers are mainly determined by flavonoids, especially anthocyanins. It is well-known that there are six main anthocyanins in plants (cyanidin, delphinidin, pelargonin, peonidin, malvidin, and petunidin). Among them, peonidin is synthesized by methylation of cyanidin and malvidin and petunidin are formed under different degrees of delphinium methylation (Martin et al., 1991; Tanaka et al., 2009). Peonidin and cyanidin appear purple-red, while pelargonin appears brick red. Delphinidin, malvidin, and petunidin are between blue and purple. Accordingly, these compounds can change the color of plants from pink to blue violet (Kazuma et al., 2003; Peng et al., 2021). Whether other non-anthocyanidins affect the different colors of flowers is an interesting topic.

**FIGURE 1:**
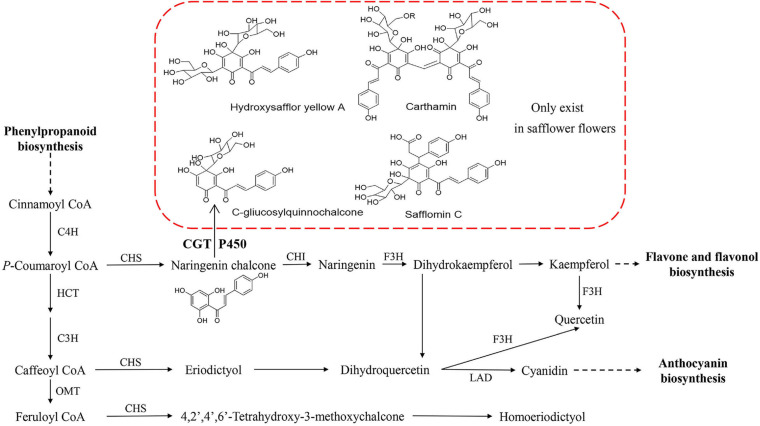
Flavonoid biosynthesis pathway in safflower flower. In red frame, some quinochalcone C-glycosides and their derivatives only existed in safflower flowers. CGT and P450 might take part in biosynthesis of HSYA. C4H, cinnamate-4-hydroxylase; HCT, shikimate O-hydroxycinnamoyltransferase; C3H, cinnamate-3-hydroxylase; OMT, O-methyltransferase; CHI, Chalcone isomerise; CHS, Chalcone synthase; F3H, Flavanone 3-hydroxylase; HYSA, Hydroxysafflor yellow A; CGT, C-glycosyltransferase; P450, cytochrome P450.

In safflower, the chemical analysis showed that more than 200 compounds were isolated, including flavonoids, alkaloids, steroids, and some other compounds (Yue et al., 2013), and flavonoids were the main metabolite species. C-glucosylquinochalcones are the main metabolic species in the red color of safflower, and they only occur in safflower (Kazuma et al., 2000). Hydroxysafflor yellow A (HSYA), also known as safflomin A, is composed of one C-glucosylquinochalcone, and it is the major component of flavonoids and responsible for the yellow pigments in the red color of safflower (Saito, 1994). Carthamin, which is composed of two C-glucosylquinochalcone moieties, responds to the red pigment of the red color of safflower (Obara and Onodera, 1979). During the long-term cultivation of safflower, many varieties present different colors of flowers, such as white (W), yellow (Y), light red (LR), and deep red (DR). Although we have a good understanding of the metabolic species in the red flowers, we know nothing of the other colors of safflower from either a chemical or molecular perspective (Figure 1).

Currently, the core flavonoid biosynthetic pathway is well-understood (Grotewold, 2006; Zhang et al., 2014; Zhao and Tao, 2015). First, phenylalanine is converted to coumaroyl-CoA by phenylalanine ammonia lyase (PAL), cinnamate-4-hydroxylase (C4H), and 4-coumarate CoA ligase (4CL), and this process is common to many secondary metabolism pathways. Second, dihydroflavonol is synthesized from coumaroyl-CoA and malonyl-CoA and catalyzed by many enzymes, such as chalcone synthase (CHS), chalcone isomerase (CHI), flavanone 3-hydroxylase (F3H), flavonoid 3′-hydroxylase (F3′H), flavonol synthase (FLS), and flavone synthase (FNS). Third, leucoanthocyanidins are formed from dihydroflavonol by the action of dihydroflavonol 4-reductase (DFR), and then the synthesis of the corresponding colored anthocyanidins by anthocyanidin synthase (ANS). Then, a series of modifications of anthocynidins are catalyzed by flavonoid glucosyltransferase (UFGT) and anthocyanin *O*-methyltransferase (AOMT) to form stable anthocyanins. Many reports have explained the flower color of plants at the molecular level based on the existing flavonoid metabolism pathway (Jin et al., 2016; Xu et al., 2017; Li et al., 2020). However, there are no one-size-fits-all rules because plants vary among each other. The metabolic species and the genes that regulate pigments are different, and it is necessary to conduct a personalized analysis for different plants.

According to the chemical structure of C-glucosylquinochalcones, C-glucosylquinochalcones and its derivatives are speculated to be formed by the glycosylation of naringin chalcone by C-glycosyltransferase (CGT) and the oxidation of cytochrome P450 (P450) (Figure 1). Although no related genes have been reported in safflower, the biosynthesis of many structural analogs has been reported in other plants, especially CGT in *Glycyrrhiza glabra* (Zhang et al., 2020) and P450 in *Glycine max* L (Latunde-Dada et al., 2001) and sweet basil (Berim and Gang, 2012), in which the reaction substrate where the enzymes participate is similar to the precursor in safflower. Their work provides a reference for us to screen candidate genes involved in C-glucosylquinochalcone biosynthesis in safflower.

In this study, to elucidate the metabolic and transcriptional differences among safflower cultivars with different-colored flowers and the genes that responded to C-glucosylquinochalcone biosynthesis, four safflower cultivars with different flower colors (W, Y, LR, and DR) were used. Metabolic, transcriptional and integration analyses were carried out among the four different safflowers. At the same time, we analyzed the expression pattern of candidate genes involved in C-glucosylquinochalcone biosynthesis, including tissue specificity expression and to the different stages of flower development. At the same time, our previous study reported that flavonoid metabolic species of safflower, especially C-glucosylquinochalcones (such as safflomin A), significantly increased after treatment with MeJA (Chen et al., 2020). We also analyzed the expression of candidate genes after treatment with methyl jasmonate (MeJA).

## Results

### Metabolic Profiling and Differential Flavonoid Metabolite Analysis Among Safflower With Different Colors

**FIGURE 2:**
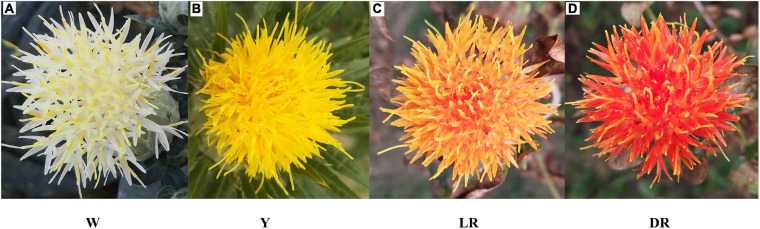
Four safflower cultivars used in this study. **(A)** The white color of safflower, W means white. **(B)** The yellow color of safflower, Y means yellow. **(C)** The light red color of safflower, LR means light red. **(D)** The deep red color of safflower, DR means deep red.

Four safflower cultivars with white (W), yellow (Y), light red (LR), and deep red (DR) flowers were used in this study (Figure 2). The secondary metabolic species were determined by a Q Exactive benchtop Orbitrap mass spectrometer. All the metabolic species are annotated through KEGG. All the raw data for the detected metabolic species are shown in Supplementary Table 1. We screened the metabolic species involved in flavonoid biosynthesis (KEGG number: ko00941 to ko00944). In addition, since C-glucosylquinochalcones, such as (safflomins A and C), are special metabolic species of safflower that were not annotated by KEGG we selected separately from the metabolite data. Finally, a total of 277 flavonoid metabolite species were identified, including 20 dihydroflavones, 22 dihydroflavonols, 26 chalcones (including C-glucosylquinochalcones), 23 aurones, 5 flavonols, 18 flavones, 22 flavonoids, 31 flavonoid carbonosides, 70 flavonols, 12 isoflavones, 10 anthocyanins, 9 phenolic acids, and 9 others. More flavonoid metabolites were detected in Y and W than in LR and DR, with 154 flavonoid metabolic species in Y and 141 flavonoid metabolic species in W, compared to 126 flavonoid metabolic species in DR and 97 flavonoid metabolic species in LR. All details are provided in Supplementary Table 2.

**FIGURE 3:**
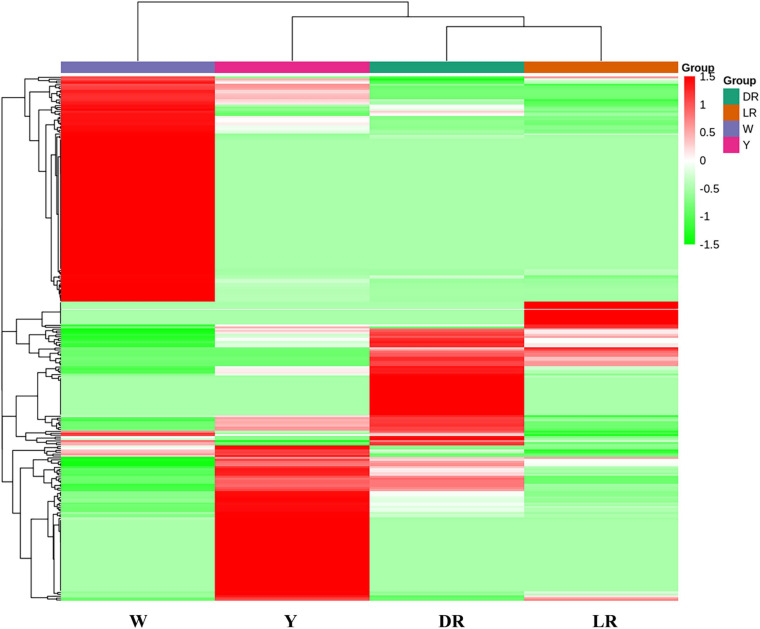
Heatmap of flavonoid metabolic species among the four different-colored flowers of safflower. The heatmap was drawn based on the content of flavonoid metabolic species in the different color of safflower. W, white; Y, yellow; LR, light red; DR, deep red.

Based on the content of flavonoid metabolic species in the different colors of safflower, a heatmap was drawn (Figure 3). The flavonoid metabolites of safflowers of different colors showed great differences, and safflowers could be distinguished well by their metabolite species, and the cluster results indicated that W was far from the other three color safflowers. To classify the different metabolite species, six groups (DR/W, LR/W, Y/W, LR/Y, DR/Y, and LR/DR) were constructed. In the DR/W group, 211 flavonoid metabolic species showed a significant difference. Eighty-eight flavonoid metabolic species were downregulated in W, mainly flavonols (23 species), chalcones (11), and dihydroflavonol (9). In addition, 123 flavonoid metabolic species were upregulated in W, mainly flavonols (34), flavonoid carbonosides (16) and aurones (12). In the LR/W group, 201 flavonoid metabolic species showed a significant difference. Sixty-eight flavonoid metabolic species were downregulated in W, and they mainly included flavonols (18), flavonoid carbonosides (10), and dihydroflavonol (9). In addition, 133 flavonoid metabolic species were upregulated in W, and they mainly included flavonols (34), flavonoid carbonosides (16), and aurones (14). In the Y/W group, 213 flavonoid metabolic species showed a significant difference. A total of 102 flavonoid metabolic species were downregulated in W, and they mainly included flavonols (32), chalcones (12), and dihydroflavonol (8). In addition, 101 flavonoid metabolic species were upregulated in W, mainly flavonols (32), flavonoid carbonosides (20), and flavones (12). In the LR/Y group, 150 flavonoid metabolic species showed a significant difference. Thirty-one flavonoid metabolic species were downregulated in Y, and they mainly included flavonoid carbonosides (6), chalcones (4), and aurones (4). In addition, 119 flavonoid metabolic species were upregulated in Y, and they mainly included flavonols (39), chalcones (15), and dihydroflavones (10). In the DR/Y group, 153 flavonoid metabolic species showed a significant difference. Fifty-four flavonoid metabolic species were downregulated in Y, and they mainly included flavonols (12), flavonoid carbonosides (9), and aurones (7). Ninety-nine flavonoid metabolic species were upregulated in Y, and they mainly included flavonols (32), dihydroflavone (9), flavonoid carbonosides (9), and aurones (9). In the LR/DR group, 114 flavonoid metabolic species showed a significant difference. Twenty-seven flavonoid metabolic species were downregulated in DR, and they mainly included flavonols (5), flavonoid carbonosides (6), and dihydroflavonol (3). Eighty-seven flavonoid metabolic species were upregulated in DR, and they mainly included flavonols (25), chalcones (11), and flavonoid carbonosides (9). The details can be viewed in Supplementary Table 3.

In addition, most flavonols and flavonoid carbonosides were more abundant in W than in the other three colors of safflowers. However, C-glucosylquinochalcones, such as safflomins A and C and their isomers, were not detected in W. Most flavonols and chalcone had a high content in Y, but the content of C-glucosylquinochalcones was lower than that in DR. With increasing color (from LR to DR), it can be clearly viewed that the content of C-glucosylquinochalcones increased. Anthocyanins were also detected in our research, and they mainly included glucosides of cyanidin, pelargonin, and delphinidin. However, there were no intersections among the detected anthocyanins. Combined with the content of C-glucosylquinochalcones, it can be concluded that the color difference among the four safflower cultivars is mainly caused by C-glucosylquinochalcones but not anthocyanins.

### Transcriptome Sequencing and Differential Transcript Analysis for the Annotated Flavonoid Biosynthesis Gene

The transcriptomes of the safflowers with four different colors were sequenced. A total of 76.04 G clean base was obtained, with 18.95 G in W, 18.54 G in Y, 19.07 G in LR and 19.48 G in DR. The average Q30 is 94.37%, with 94.32% in W, 94.34% in Y, 94.39% in LR and 94.44% in DR. The raw data was submitted to NCBI (BioProject ID: PRJNA738310). Through gene annotation, a total of 22,432 genes were annotated in Trembl, 19,108 genes were annotated in GO, 18,075 genes were annotated in KEGG, and 22,643 genes were annotated in NR (Supplementary Table 4). In this study, RNAseq were validated by qPCR, and the results of RNAseq were almost consistent with the results of qPCR, indicating the accuracy of sequencing data (Supplementary Figure 1). The differentially expressed genes were analyzed with DESeq2. A total of 9,997 genes showed different expression levels (Supplementary Table 5). To classify the differentially expressed genes, six groups (LR/W, DR/W, W/Y, LR/Y, DR/Y, and LR/DR) were also constructed. In the LR/DR group, 2,759 genes were differentially expressed. In the LR/W group, 2,373 genes were differentially expressed. In the LR/Y group, 2,079 genes were differentially expressed. In group DR/W, 4,315 genes were differentially expressed. In group DR/Y, 2,271 genes were differentially expressed. In group W/Y, 1,794 genes were differentially expressed.

**FIGURE 4:**
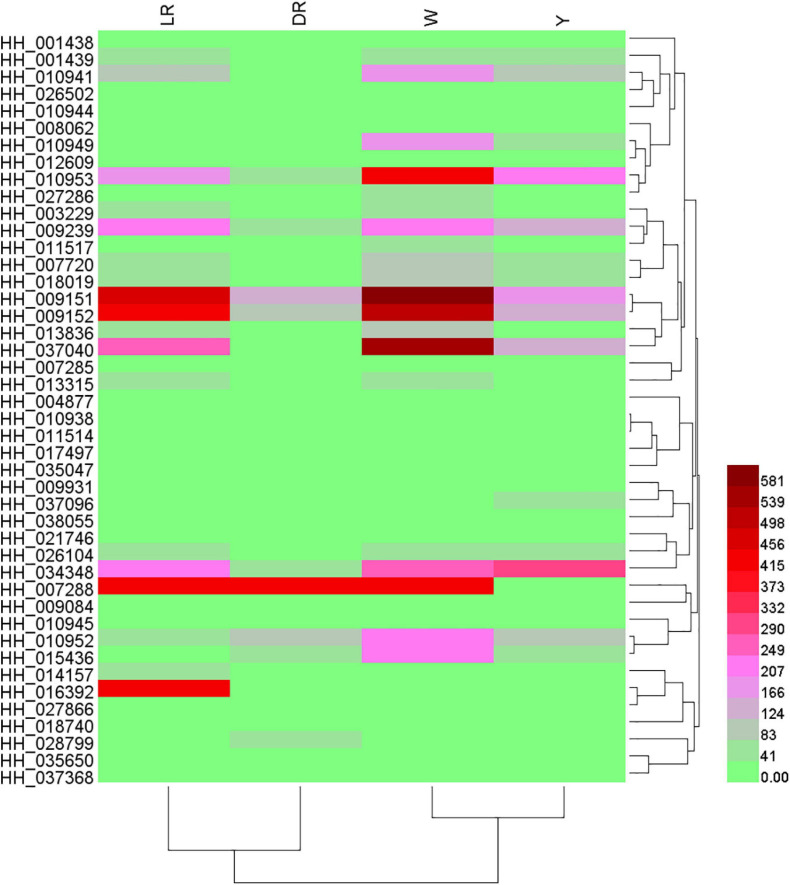
Heatmap of annotated flavonoid biosynthesis genes among the four different-colored flower of safflower. The heatmap was drawn based on relative expression of annotated flavonoid biosynthesis genes. W, white; Y, yellow; LR, light red; DR, deep red.

We focused on the genes participating in the biosynthesis of flavonoids. From the results of the differential transcript analysis, a total of 44 flavonoid biosynthesis genes were screened, and the details of their expression can be viewed in Supplementary Table 6. Based on their expression, a heatmap were drawn (Figure 4). There was a huge difference in the gene expression. In the DR/W group, 29 genes showed different expression, and most of the flavonoid pathway genes, including isoflavone biosynthesis and flavonol and flavonol biosynthesis genes, were significantly regulated in W, such as CHS (HH_007720, HH_018019), shikimate O-hydroxycinnamoyltransferase (HCT) (HH_001438, HH_026502), OMT (HH_008062), F3H (HH_009239), and vestitone reductase (VR) (HH_015436). In the LR/W group, 18 genes showed different expression; similarly, most of the flavonoid pathway genes were upregulated, such as OMT (HH_008062), AS (HH_027286), anthocyanidin 3-O-glucoside 6-O-acyltransferase (AOAT) (HH_010944, HH_010945), and VR (HH_015436), whereas several genes showed a downregulated expression pattern, such as trans-cinnamate 4-monooxygenase (HH_014157), HCT (HH_009931, HH_016392), CHI (HH_027866), flavone synthase II (FSII) (HH_018740), DFR (HH_037096), flavone synthase (FS) (HH_035650), and ANR (HH_038055). In the W/Y group, 16 genes showed different expression, and most flavonoid biosynthesis genes were upregulated, such as CHI (HH_027866), DFR (HH_037096), 2-hydroxyisoflavanone dehydratase (HIDH) (HH_035047), and AOAT (HH_010938), while some flavonoid biosynthesis genes were downregulated, such as CHS. In the DR/Y group, 16 genes showed different expression, and most flavonoid biosynthesis genes were upregulated, such as CHS (HH_018019), CHI (HH_011517), ANR (HH_038055), HCT (HH_017497), 2-hydroxyisoflavanone dehydratase (HIDH) (HH_035047), isoflavone 7-O-glucoside-6-O-malonyltransferase (IF7MAT) (HH_010949), VR (HH_026104), and AOAT (HH_010944). In the LR/Y group, 17 genes showed different expression, and some flavonoid biosynthesis genes were downregulated, such as CHI (HH_027866) and shikimate O-hydroxycinnamoyltransferase (HH_007288, HH_016392), while some flavonoid biosynthesis genes were upregulated, such as caffeoyl-CoA O-methyltransferase (HH_008062), 2-hydroxyisoflavanone dehydratase (HH_035047), VR (HH_015436), and AOAT (HH_010938). In the LR/DR group, 21 genes showed different expression, and most flavonoid biosynthesis genes were significantly downregulated, such CHS (HH_007720, HH_HH_018019), HCT (HH_001438, HH_016392), naringenin 3-dioxygenase (HH_009239), DFR bifunctional dihydroflavonol 4-reductase/flavanone 4-reductase (HH_037096), ANR (HH_038055), CHI (HH_027866), 3AT (HH_010941), and VR (HH_026104).

In general, most of the annotated flavonoid synthesis genes were significantly increased in W compared to those in LR or DR. With increasing color, the expression of annotated flavonoid biosynthesis was decreased. The expression of the most annotated flavonoid genes was the lowest in DR, while the expression of the most annotated flavonoid genes was the highest in W.

### Transcriptome Sequencing and Differential Transcript Analysis of the Candidate Genes Involved in C-Glucosylquinochalcones

From the above results, the expression levels of the annotated flavonoid synthesis genes in DR were very low, confirming that these genes were not responsible for the red or yellow color of safflower. Considering that biosynthesis of C-glucosylquinochalcones is not annotated in KEGG, and combined with the structure of C-glucosylquinochalcones and reports in *Glycyrrhiza glabra* (Zhang et al., 2020), *Glycine max* L (Latunde-Dada et al., 2001), sweet basil (Berim and Gang, 2012), two genes of UGTs (Cluster-1824.147334 and BAR73279.1, ID in the report of Zhang et al., 2020) and two genes of P450 (tr| M1KVN4| MENPI and cytochrome P450 71D9, ID in the report of Latunde-Dada et al., 2001; Berim and Gang, 2012) were chosen as the target genes to blast with the safflower genome in our lab (unpublished) to screen the candidate genes involved in C-glucosylquinochalcone biosynthesis. We chose the top 10 candidate genes for each target gene with high similarity for further analysis. All the details of the target genes and the candidate genes can be viewed in Supplementary Table 7.

**FIGURE 5:**
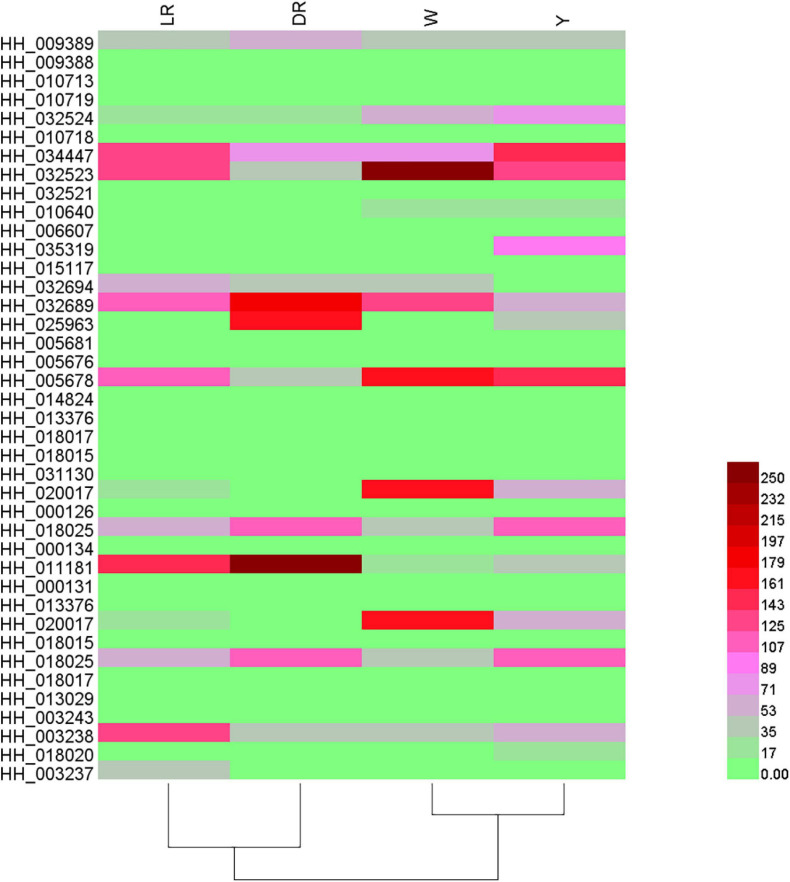
Heatmap of gene expressions of the candidate genes involved in C-glucosylquinochalcones. The heatmap was drawn based on relative expression of the candidate genes involved in C-glucosylquinochalcones biosynthesis. W, white; Y, yellow; LR, light red; DR, deep red.

The expression patterns of the candidate genes among the different colors of safflower were analyzed. For the UGT homologous genes, HH_018017, HH_018017, and HH_013029 showed no expression in the flowers, and HH_013376, HH_018015, HH_031130, HH_000134, HH_000131, HH_013376, and HH_018015 showed slight expression in the flowers. Three homologs to Cluster-1824.147334 (HH_018025, HH_011181, HH_000131) and two homologs to BAR73279.1 (HH_003243, HH_003238) showed major expression in flowers. For the P450 homologous genes, HH_009388, HH_032521, and HH_005676 showed no expression in the flowers. HH_010713, HH_010718, HH_015117, and HH_014824 showed little expression in the flowers, three genes homologous to tr| M1KVN4| MENPI (HH_009389, HH_010713, HH_034447) and three homologous to cytochrome P450 71D9 (HH_035319, HH_032689, HH_025963) showed major expression in flowers. The heatmap of the gene expression is shown in Figure 5.

### Integrated Analysis of the Transcriptome and Metabolome of the Flavonoid Biosynthesis Pathway With Different Colors

**FIGURE 6:**
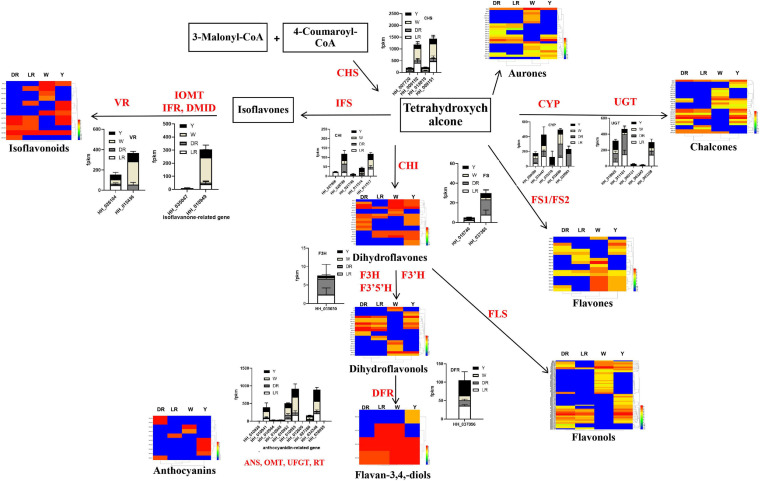
Integrated analysis of the transcriptome and metabolome on the flavonoid biosynthesis pathway with different colors. The core schematic diagram of flavonoid synthesis in safflower was drawn based on our previous research reports (Chen et al., 2020) and the review of Winkel-Shirley (2002). Various flavonoid metabolic species detected in safflower and the annotated flavonoid biosynthesis genes and the candidate genes for biosynthesis of C-glucosylquinochalcones were shown in the figure. Gene expression was represented by stacked graphs and that the length of each bar shows the expression of each gene. DFR, dihydroflflavonol 4-reductase; IFS, Isoflavone Synthase; OMT, O-methyltransferase; CHI, Chalcone isomerise; CHS, Chalcone synthase; F3H, Flavanone 3-hydroxylase; VR, vestitone reductase; CGT, C-glycosyltransferase; P450, cytochrome P450; FLS, flavonol synthase.

Based on our previous research reports (Chen et al., 2020) and the review of Winkel-Shirley (2002), we drew the core schematic diagram of flavonoid synthesis in safflower. Various flavonoid metabolic species detected in safflower were mapped in the form of a heatmap, and the heatmap for the different types of flavonoids can be viewed in the Supplementary Figure 2. At the same time, we classified the different flavonoid genes among the different colors of safflower and constructed a bar chart of the expression of different genes. For the candidate genes for C-glucosylquinochalcone biosynthesis, we selected the genes once they had expression in flowers, especially in red flowers. All the metabolic species and the gene expression levels were integrated into the schematic diagram (Figure 6).

CHS is a rate-limiting enzyme in the biosynthesis of flavonoids. From the results, it can be clearly viewed that the expression of different expressed CHS genes was the highest in W, and the higher in LR. Considering that both the number and content of flavonoid metabolic species were higher in W, this may be caused by the high expression of CHS genes. In addition, CHI is another key enzyme in the biosynthesis of flavonoids. The expression of CHI (HH-028799) in DR was relatively high. Combined with the metabolomics results, the high content of the metabolic species detected in DR (MW-217, MW-246) may be due to this gene. During the biosynthesis of isoflavones, the expression levels of HH-015436 and HH-010949 were relatively high. There were several flavonoid metabolic species, especially those detected in W, and were also detected in other flowers and colors, whose content in W was relatively high, which may be due to the genes of HH-015436 and HH-010949. To the biosynthesis of C-glucosylquinochalcones, we can see that several genes, such as HH_034447 and HH_025963 for P450 and HH_011181 HH_000131 and HH_011181 for CGT, had an expression in DR and LR. Combined with the detected C-glucosylquinochalcones, it is possible that these genes are involved in the biosynthesis of C-glucosylquinochalcones in red flowers. There is also much more information to be mined from the integrated metabolic schematic diagram, which provides clues for screening the genes responsible for the biosynthesis of flavonoids in each color flower.

### Quantitative Analysis of the Candidate Genes Involved in Safflower C-Glucosylquinochalcone Biosynthesis

**FIGURE 7:**
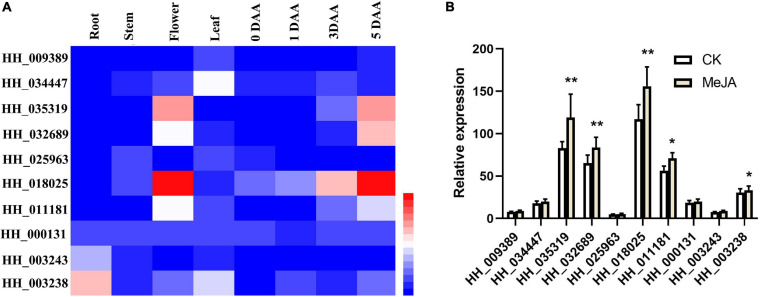
Quantitative analysis of the candidate genes of C-glucosylquinochalcones biosynthesis. **(A)** The heatmap of the expression of candidate genes, based on the different tissues and different stages of flower development. **(B)** The bar graph of the candidate genes after the treatment with MeJA. **P = 0.01, *P = 0.05.

To further screen candidate genes involved in C-glucosylquinochalcone biosynthesis, we first analyzed the expression pattern of the candidate genes that had expression in flowers (Figure 7A). It is known that the biosynthesis of C-glucosylquinochalcones in safflower is mainly expressed in flowers and mainly accumulates during the flowering stage. In this way, genes that have similar patterns to the accumulation of C-glucosylquinochalcones are more likely to be involved in C-glucosylquinochalcone biosynthesis. HH_035319, HH_032689, HH_018025, and HH_011181 were mainly expressed in flowers, HH_003243 and HH_003238 were mainly expressed in roots, and HH_034447 was mainly expressed in leaves. Except for these genes, the expression of other genes was low. In addition, with the development of flowers, the expression of HH_035319, HH_032689, and HH_018025 HH_011181 significantly increased, while the other genes were not obvious, such as HH_009389, HH_025963, and HH_003243, which basically did not increase, and these findings indicated that HH_035319, HH_032689, and HH_018025 HH_011181 might participate in the biosynthesis of C-glucosylquinochalcones.

At the same time, our previous study reported that flavonoid metabolic species of safflower, especially C-glucosylquinochalcones (such as safflomin A), significantly increased after treatment with MeJA (Chen et al., 2020). Therefore, we speculated that the functional genes involved in C-glucosylquinochalcone biosynthesis should also be upregulated by MeJA. The candidate gene expression in response to MeJA (Figure 7B) was determined. The results showed that HH_035319, HH_032689, and HH_018025 showed a significant increase after the MeJA treatment (*P* = 0.01). HH_011181 and HH_003238 also significantly increased during MeJA treatment (*P* = 0.01). Overall, the results indicated that HH_035319, HH_032689, and HH_018025 might participate in the biosynthesis of C-glucosylquinochalcones, which ultimately leads to the red color in safflower.

## Discussion

Flavonoids are the main metabolic species in safflower, and a total of 277 metabolic species were detected and annotated as flavonoids by KEGG, which is far more than our previous research, in which 209 flavonoid metabolites were detected based on a triple quadrupole-linear ion trap mass spectrometer (Q TRAP), API 6500 QTRAP LC/MS/MS system equipped with an ESI Turbo ion-spray interface (Chen et al., 2020). Although a number of isomers may be involved in the identification of metabolite species, different metabolic species are observed by the time the peak is observed in the liquid phase; thus, we confirmed that 277 components were detected. Since C-glucosylquinochalconesis was not included in the classification, such as safflomin A and safflomin C and their isomerides, it was selected separately. In this way, we can see the overall number and content of flavonoid metabolic species in safflower.

There are six main anthocyanins in plants: cyanidin, delphinidin, pelargonin, peonidin, malvidin, and petunidin, which can change the color of plants from pink to blue violet (Kazuma et al., 2003; Peng et al., 2021). Anthocyanins were detected in this study, mainly including the glucosides of cyanidin, pelargonin, and delphinidin. However, there were no overlap among the detected anthocyanins. C-glucosylquinochalcones, such as safflomins A and C and their isomers, which were not detected in W. As the color increased, such as from LR to DR, the content of C-glucosylquinochalcones clearly increased. The results show that the color difference is mainly caused by C-glucosylquinochalcones.

The combination of different omics techniques provides a deeper understanding of several crucial genes involved in plant growth, development, and different stress responses (Moreno-Risueno et al., 2010; Casimiro-Soriguer et al., 2016; Wang et al., 2020). In safflower, studies have used different types of safflower to elucidate the details on color biosynthesis. Qiang et al. (2020) used transcriptome sequencing and chemical analysis to reveal the formation mechanism of white florets in safflower. However, the pigment contents of red and white flowers were determined only using a spectrophotometer. The material basis of the chemical composition between the different colors is largely unknown. Through the transcriptome analysis, only the synthesis pathway of the common flavonoid biosynthesis pathway was considered. In our results, there were more flavonoid species in Y and W, and the same flavonoid composition had higher expression in LR and W. *CHS* is the key gene in flavonoid biosynthesis, Expression analysis show that it was more highly expressed in W and LR than in other flowers and colors, which may explain the high flavonoid types and content in W and LR. At the same time, there was no one-to-one correlation between some genes and flavonoid metabolic species from the comparison of the existing flavonoid metabolic species and the annotated genes, which may be related to the lack of identification of a number of genes involved in flavonoid biosynthesis in safflower. This subject is worthy of further study. Of course, when mining the genes participating in flavonoid biosynthesis, our results can be favored, as we provided a lot of data about the candidate flavonoid biosynthesis genes and the related flavonoid species.

The chemical structure of C-glucosylquinochalcones indicates that it is formed by the glycosylation of naringin chalcone by C-glycosyltransferase (CGT) and the oxidation of cytochrome P450 (P450). At present, there are also a few reports on glycosyltransferases in safflower (Xie et al., 2014), although only one nitrogen-bonded glycosyltransferase has been identified, and it catalyzes N-nitroglucoside, O-oxyglucoside, and S-glucoside and not C-carbonoside. Our previous study reported that flavonoid metabolic species of safflower, especially C-glucosylquinochalcones (such as safflomin A), significantly increased after treatment with MeJA (Chen et al., 2020). Based on the expression pattern and their response to MeJA treatment, three candidate genes, HH_035319, HH_032689, and HH_018025, likely take part in the biosynthesis of C-glucosylquinochalcones. In our future research, these genes will be cloned and analyzed.

## Materials and Methods

### Plant Materials

Four kinds of safflower materials with different colors, white (W), yellow (Y), light red (LR), and deep red (DR), were used in this study. They were cultivated at the medicinal botanical garden on the Wenjiang Campus of Chengdu University of Traditional Chinese Medicine. For RNA sequencing, five inflorescences of safflower were mixed as a sample and three replicates for RNA sequencing were carried out. For the metabolism analysis, ten inflorescences of safflower plants were mixed as one sample.

### Liquid Chromatography and Mass Spectrometry

The freeze-dried sample was crushed using a mixer mill (MM 400, Retsch) with a zirconia bead for 1.5 min at 30 Hz. One hundred milligrams of powder was weighed and extracted overnight at 4°C with 1.2 mL 70% aqueous methanol. Following centrifugation at 12 000 rpm for 10 min, the extracts were filtered and used for liquid chromatography and mass spectrometry analyses. A Thermo Scientific^TM^ UltiMate 3000 UHPLC and Q Exactive benchtop Orbitrap mass spectrometer were used for data acquisition It has been widely used for metabolic species detection (Wang et al., 2012; De Paepe et al., 2019). The chromatography analysis was carried out by a C18 column (Thermo Scientific Accucore^TM^ 4.6 mm × 100 mm, 1.8 μm). The mobile phase was eluted with 0.1% formic acid water (A) to 0.1% acetonitrile (B) by gradient elution (0–3 min, 5% B; 3–10 min, 5–15% B; 10–25 min, 15–25% B; 25–35 min, 25–85% B; 35–40 min, 85% B; 40–43 min, 85–5% B; 43–50 min, 5% B), the flow rate was 0.2 mL⋅min^–1^, the column temperature was 35°C, and the injection volume was 3 μL. An electrospray ion source (ESI) was used for positive ion detection. The spray voltage was 3.2 kV, the ion source temperature was 350°C, the sheath gas flow rate was 35 arb, the auxiliary gas flow rate was 10 arb, and the ion transport tube temperature was 320°C. The scanning mode was full MS/DD-MS2, with a first-level resolution of 35,000 and a second-level resolution of 17,500. The scanning range was m/z 100–1,500, and the impact energy gradient was 20, 40, and 60 eV.

### Data Analysis

The raw data were imported to Compound Discoverer 3.0 software through template wizard settings and methods to establish the unknown compound identification process. Peak alignment and peak extraction were performed on the original data. The possible molecular formula can be fitted by the molecular ion chromatographic peak and isotope peak obtained from the extraction. The measured spectrum of secondary fragments was matched with the MZCloud network database and local TCM component database OTCML. The filtering parameters for matching results were set as follows: peak area threshold 80,000, the quality of primary and secondary deviation 5 ppm. The matching score was higher than 80. Compounds were analyzed and identified by comparing the filtered ions with the compound information in the database, reference substances, and related literature. The identified metabolic species were annotated using the KEGG Compound database^2^, and annotated metabolic species were then mapped to the KEGG Pathway database^3^. We focused on the analysis of flavonoids. The metabolic species annotated through KEGG (KEGG number: ko00941 to ko00944) were treated as flavonoids. In addition, as C-glucosylquinochalcone was not annotated in KEGG, C-glucosylquinochalcones were selected separately, together with the notes of flavonoids, as the analysis object.

Unsupervised PCA (principal component analysis) was performed by statistical analysis within R^4^. The data were unit variance-scaled before unsupervised PCA. The HCA (hierarchical cluster analysis) results of the samples and metabolites are presented as heatmaps with dendrograms, while the Pearson correlation coefficients (PCCs) between samples were calculated by the cor function in R and presented as only heatmaps. Both HCA and PCC were carried out by R package pheatmap. For HCA, the normalized signal intensities of metabolites (unit variance scaling) were visualized as a color spectrum. Significantly regulated metabolites between groups were determined by VIP = 1 and absolute Log_2_ FC (fold change) = 1. VIP values were extracted from the OPLS-DA results, which also contained score plots and permutation plots and were generated using the R package MetaboAnalyst R. The data were log transformed (log2) and mean centered before OPLS-DA. To avoid overfitting, a permutation test (200 permutations) was performed.

### RNA Sequencing and Annotation

RNA isolation and purification and cDNA library construction and sequencing were performed as previously described (Wang et al., 2017). All tissues were ground on dry ice, and total RNA was prepared using TRIzol reagent (Invitrogen, CA, United States). To remove DNA, an aliquot of total RNA was treated with DNase (Takara, Dalian, China). The RNA quantity and quality were determined using a NanoDrop 2000 spectrophotometer (NanoDrop Technologies, Wilmington, DE, United States) and an Agilent 2100 Bioanalyzer (Agilent Technologies, CA, United States), respectively. mRNA was isolated from total RNA using magnetic beads with oligo (dT) primers; cDNA was synthesized using a cDNA synthesis kit (TaKaRa, Dalian, China) and linking the sequencing adapter to both ends. The library preparations were sequenced on an Illumina HiSeq 4000 platform, and clean reads after quality control were compared to the reference genome (not published). Clean reads were sequenced with the reference genome by HisAT2 to obtain position information on the reference genome or gene as well as specific sequence characteristic information of the sequenced samples.

### Screening of Differential Genes

DESeq2 (Love et al., 2014; Varet et al., 2016) was used for differential expression analysis among sample groups. DESeq2 requires the input of unregulated read counting data of genes rather than RPKM (reads per kilobase million), FPKM (fragments per kilobase million) and other standardized data. The read counts of genes are the expected count outputs calculated using RSEM (RNAseq by Expectation-Maximization). The expected count is generally lower than the read number. After the discrepancy analysis, multiple hypothesis tests are needed to correct the hypothesis test probability (*P*-value) with the Benjamini-Hochberg procedure to obtain the false discovery rate (FDR) when | log_2__–_fold change| ≥ 1 and FDR < 0.05.

### Validation of RNAseq by qPCR

The results of RNAseq should be validated by qPCR to see if there is a good correlation between the expression obtained by both techniques. In this study, five genes (HH_000029, HH_000060, HH_000086, HH_000142, and HH_000164) were randomly selected from RNAseq to make the validation. Firstly, the primers for the qPCR were designed quantitative primers using Primer 5, F: ACACCGCAAGAACAACAA and R:CACCTGAGCCGTAAGAAA were used as forward and reverse primers for HH_000029. F:GCTAATTTGCTAAT CCGTAC and R:GTGCCTCAGAAGACACTC were used as forward and reverse primers for HH_000060. F:TGCTGCTTTCTCCTCACA and R:ATTAGGCTCGC TCCATAC were used as forward and reverse primers for HH_000086. F:TCTTGTGGCTGTTGTGATTG and R:AGAGGCATTTCGGGTTTT were used as forward and reverse primers for HH_000142. F:AGCCATTGCTCCTTCTGA and R:AACCCTTCCTTGGTCTGC were used as forward and reverse primers for HH_000164. The qPCR analysis was performed using Bio-Rad CFX96 system (Bio-Rad, CA, United States) in a total reaction volume of 25 μL of SYBR Green PCR Master Mix (TaKaRa). The Ct value was determined using the instrument’s software. The relative quantification of gene expression was monitored after normalization to *28S* rRNA expression as internal control. F:GGGTCCTTTCACGTTTCTGA and R: GGCCTGACTTATCGGTAGCA used as forward and reverse primers for *28S*. The relative transcription levels were calculated using the ΔCt method. We compared the gene expression of RNAseq data (based on FPKM) with qPCR data. The accuracy of RNAseq was judged by analyzing the expression similarity between the two data. The details can be viewed in Supplementary Figure 1.

### MeJA Treatment

The treatment was primarily applied according to our previous report with some modifications (Chen et al., 2020). A 100 μM solution of MeJA (Sigma-Aldrich, Switzerland) was sprayed onto healthy safflower flowers 3 days after anthesis (DAA). In the control group, the flowers were sprayed with the same solution but without MeJA. The flowers were then enclosed in clear plastic bags to prevent the emission of volatile phytohormones and to allow for the elicit or solutions to be more highly absorbed. After treatment for 6 h, the plastic bags were removed and samples of flowers were collected, frozen immediately in liquid nitrogen and stored in a freezer at –80°C. Five inflorescences of safflower were mixed as a sample and used for the RNA extraction. Reverse transcription into cDNA was then used for real-time PCR analysis.

### Real-Time PCR Analysis

Real-time PCR analysis was performed as previously described (Chen et al., 2018). Total RNA was isolated using an RNA extraction kit (Invitrogen, CA, United States), and reverse transcription was carried out using the Prime Script Reagent Kit (Takara, Dalian, China). The primers used to amplify the screened genes (CHS, CHI, HCT, etc.) by real-time PCR were designed by Primer 5.0, and parts of the safflower *28S* coding region were used as an internal reference gene. The primer details are listed in Supplementary Table 7. All of the primers were tested for their specificity by agarose gel electrophoresis. RT-PCR was performed using a SYBR prime script RT-PCR kit (TaKaRa, Dalian, China) with three replicates, and the cycling conditions were set according to the manual. The Bio-Rad CFX96 real-time PCR detection system (Hercules, CA, United States) was used in our experiment.

## Data Availability Statement

The original contributions presented in the study are publicly available. This data can be found here: National Center for Biotechnology Information (NCBI) BioProject database under accession number PRJNA738310.

## Author Contributions

JC and QW conceived and designed the experiments and wrote the manuscript. RW, SD, CR, and BX performed the experiments. CC contributed to the material planting and sample collection. JW and JP contributed to the data analysis. All authors discussed the results, commented on the manuscript, and read and approved the final manuscript.

## Conflict of Interest

The authors declare that the research was conducted in the absence of any commercial or financial relationships that could be construed as a potential conflict of interest.

## Publisher's Note

All claims expressed in this article are solely those of the authors and do not necessarily represent those of their affiliated organizations, or those of the publisher, the editors and the reviewers. Any product that may be evaluated in this article, or claim that may be made by its manufacturer, is not guaranteed or endorsed by the publisher.

## Footnotes

1 http://www.fao.org/faostat/en/#home

2 http://www.kegg.jp/kegg/compound/

3 http://www.kegg.jp/kegg/pathway.html

4 www.r-project.org

## Supplementary Material

Supplementary Figure 1 Validation of RNAseq by qPCR. **(A)** The RNA detection of the sample returned. **(B)** Gene expression from the RNAseq data (based on FPKM). **(C)** Gene expression from qPCR data (based on ΔCt method).

Supplementary Figure 2 Heatmap for the different types of flavonoids among the different color of safflower. W, white; Y, yellow, LR, light red; DR, deep red.

Supplementary Table 1 The raw data for the detected metabolic species in our manuscript.

Supplementary Table 2 Details of flavonoid metabolic species in four different color flowers of safflower.

Supplementary Table 3 Differential flavonoid metabolic species among the different color flower of safflower.

Supplementary Table 4 Details of all the gene annotation.

Supplementary Table 5 Details of all the different expression genes.

Supplementary Table 6 Details of the different expression of flavonoid biosynthesis genes in the four different color flowers of safflower.

Supplementary Table 7 Details of the target genes and the candidate genes for the biosynthesis of C-glucosylquinochalcones.
