# Supplementary figures and images for: Integrated Metabolomics and Transcriptome Analysis of Flavonoid Biosynthesis in Safflower (Carthamus tinctorius L.) With Different Colors
Source: Front Plant Sci. 2021 Jul 26;12:712038. doi: 10.3389/fpls.2021.712038 (PMC8351732; doi:10.3389/fpls.2021.712038)

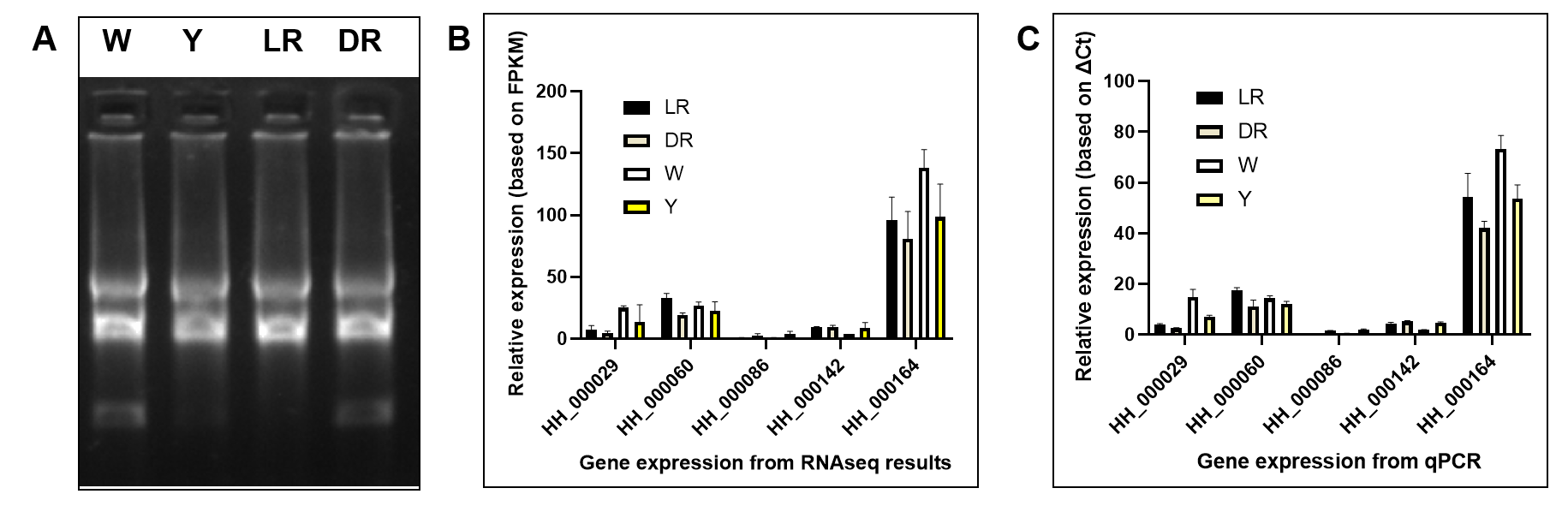

Supplement: Supplementary Figure 1 — Validation of RNAseq by qPCR. (A) The RNA detection of the sample returned. (B) Gene expression from the RNAseq data (based on FPKM). (C) Gene expression from qPCR data (based on ΔCt method). [file Image_1.TIF]

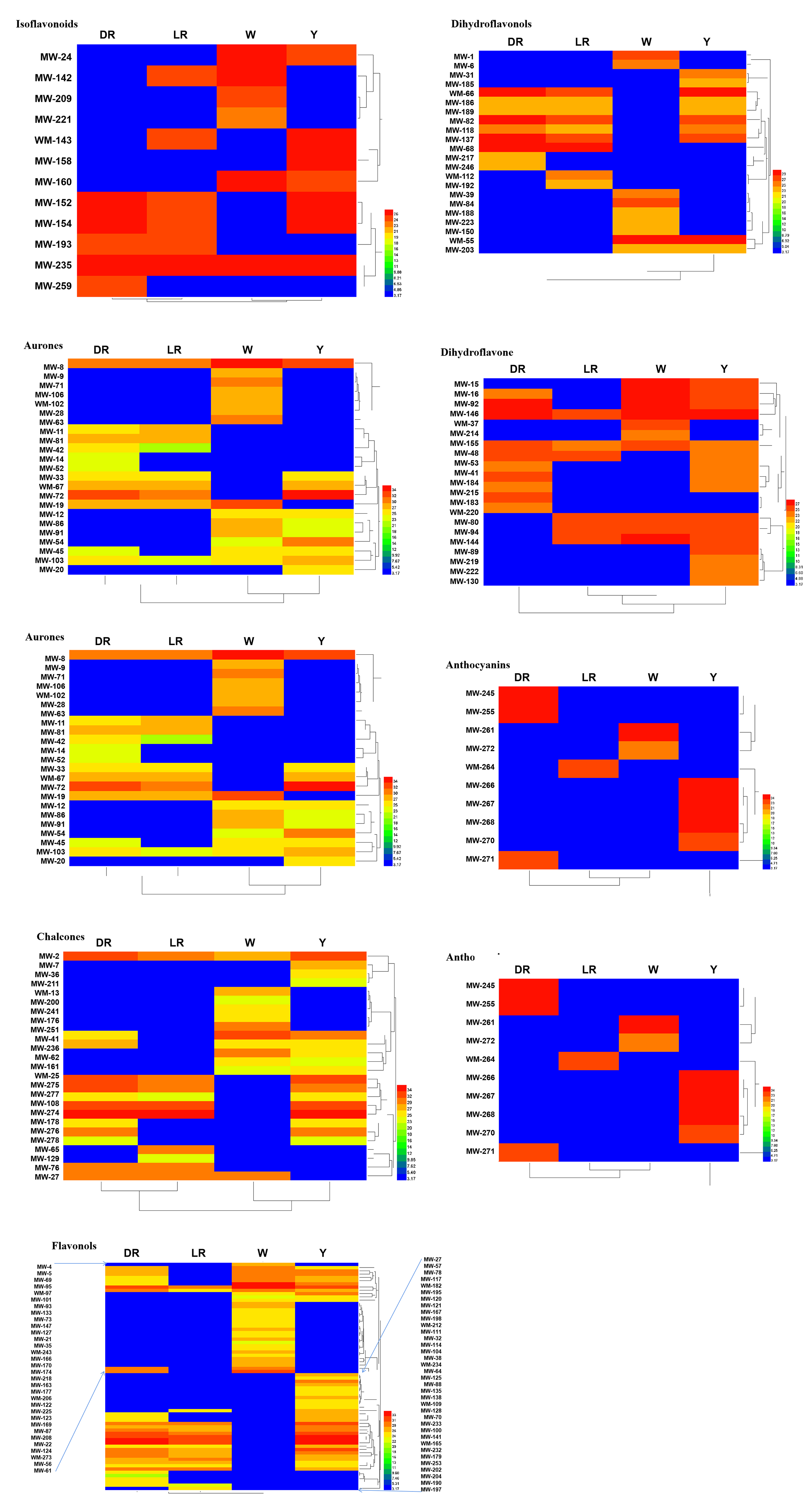

Supplement: Supplementary Figure 2 — Heatmap for the different types of flavonoids among the different color of safflower. W, white; Y, yellow, LR, light red; DR, deep red. [file Image_2.TIF]
